# Change over time in ability to perform activities of daily living in myotonic dystrophy type 1

**DOI:** 10.1007/s00415-020-09970-6

**Published:** 2020-06-15

**Authors:** Erik Landfeldt, Nikoletta Nikolenko, Cecilia Jimenez-Moreno, Sarah Cumming, Darren G. Monckton, Catharina G. Faber, Ingemar S. J. Merkies, Grainne Gorman, Chris Turner, Hanns Lochmüller

**Affiliations:** 1 Department of Women’s and Children’s Health, Karolinska Institutet, Karolinska Vägen 37A, 171 76 Stockholm, Sweden; 2 National Hospital for Neurology and Neurosurgery, Queen Square, University College London Hospitals NHS Foundation Trust, London, UK; 3 Welcome Center for Mitochondrial Research, Translational and Clinical Research Institute, Newcastle University, Newcastle upon Tyne, UK; 4 Patient-Centered Research, Evidera, London, UK; 5 Institute of Molecular, Cell and Systems Biology, College of Medical, Veterinary and Life Sciences, University of Glasgow, Glasgow, UK; 6 Department of Neurology, School of Mental Health and Neuroscience, Maastricht University Medical Center, Maastricht, The Netherlands; 7 Department of Neurology, Curaçao Medical Centre, Willemstad, Curaçao; 8 Institute of Neuroscience, Wellcome Trust Centre for Mitochondrial Research, University of Newcastle, Newcastle, UK; 9 Queen Square Department of Neuromuscular Disease, University College London, London, UK; 10 Department of Neuropediatrics and Muscle Disorders, Medical Centre, Faculty of Medicine, University of Freiburg, Freiburg, Germany; 11 Children’s Hospital of Eastern Ontario Research Institute; Division of Neurology, Department of Medicine, The Ottawa Hospital; and Brain and Mind Research Institute, University of Ottawa, Ottawa, Canada

**Keywords:** Disability, Participation, Activities of daily living, PhenoDM1

## Abstract

**Objective:**

The objective of this longitudinal, observational study was to investigate change over time in ability to perform activities of daily living in myotonic dystrophy type 1 (DM1).

**Methods:**

Adults with genetically confirmed DM1 were recruited as part of the PhenoDM1 study in the UK. Data on activities of daily living were recorded through the DM1-Activ^C^ at baseline and a follow-up visit after 12 (± 3) months. A subset of patients had advanced genetic testing to determine the size of the progenitor allele.

**Results:**

Our sample comprised 150 patients with DM1 (mean age: 45 years; 52% female). Mean follow-up was 383 days. Mean DM1-Activ^C^ total score at baseline was 71.24 (95% confidence interval 67.77–74.71) and at the follow-up visit 69.04 (65.54–72.54). Approximately 43% of patients had a lower score at the follow-up visit (indicating a decreased ability to perform activities of daily living), 24% a higher score (indicating an increased ability), and 33% the same score at baseline and follow-up. The mean annual change in the DM1-Activ^C^ total score, estimated at − 2.06 (− 3.54 to − 0.59), was significantly related to patients’ baseline score, but not sex, disease duration, timed test results, or cytosine-thymine-guanine repeat length.

**Conclusions:**

Change over time in ability to perform activities of daily living as recorded through the DM1-Activ^C^ varies substantially between patients with DM1. Our data contribute to the understanding of the natural evolution of the disease, and should be helpful to inform the design of future trials based on the DM1-Activ^C^.

**Electronic supplementary material:**

The online version of this article (10.1007/s00415-020-09970-6) contains supplementary material, which is available to authorized users.

## Introduction

Myotonic dystrophy type 1 (DM1) is a progressive, yet highly heterogeneous multi-system disorder affecting muscle strength and mobility, amongst many other clinical domains [1]. Individually or collectively, these disease manifestations lead to impaired performance in daily life and restriction in social participation [2, 3]. The clinical variability of DM1, which partly has been attributed to the nature of the underlying gene defect as expressed via unstable triplet repeats of the *DMPK* gene, makes the design of clinical trials in this indication challenging, particularly the identification and selection of outcome measures that are relevant and fit for purpose to measure drug benefits across the full spectrum of disease severity and morbidity [4, 5].

The DM1 activity and participation scale for clinical use (DM1-Activ^C^) is an outcome tool developed to measure ability to perform activities of daily living (e.g., taking a shower, visiting family or friends, and walking up a flight of stairs) in patients with DM1 [6, 7]. It was designated as the primary endpoint in a recently completed clinical trial of cognitive behavioral therapy with optional graded exercise therapy in patients with DM1 (i.e., the OPTIMISTIC trial) [8]. However, to date, no study has in detail examined changes over time in outcomes from the DM1-Activ^C^ aside changes in total scale scores in patients with DM1. Accordingly, the objective of this study was to investigate natural changes over time in ability to perform activities of daily living as recorded using the DM1-Activ^C^ in patients with DM1 in the UK receiving no specific treatment. A specific aim was to examine the association between perceived performance at baseline, sex, disease duration, and cytosine-thymine-guanine (CTG) repeat length, and change across follow-up, respectively.

## Methods

### Study design and patient sample

This was a longitudinal, observational study of patients with genetically confirmed DM1 recruited from Newcastle University (Newcastle upon Tyne, UK) and University College London Hospitals NHS Foundation Trust (London, UK) as part of the Myotonic Dystrophy Type 1 Deep Phenotyping to Improve Delivery of Personalized Medicine and Assist in the Planning, Design and Recruitment of Clinical Trials (PhenoDM1) study (ClinicalTrials.gov identifier: NCT02831504). The following inclusion criteria were imposed for patient study eligibility: (1) ≥ 18 years of age, (2) genetically confirmed diagnosis of DM1, and (3) ability to perform the 10 m walking test at selected pace without any assistance (walking devices allowed). All participants provided informed consent to participate in the study and ethical approval was granted by the Newcastle and North Tyneside Ethics Committee (reference: NE/15/0178).

### Study procedures and outcomes

Eligible patients were asked to complete the DM1-Activ^C^ as part of the study visits. The DM1-Activ^C^ was initially developed in 2010 [6], but re-constructed in 2015 [7], and the current version encompasses a total of 25 items, each described in three levels. The tool has been developed using modern psychometric analysis (i.e., Rasch analysis [9]) and has been shown to adhere to the epistemological requirements for stable measures, including linearity, invariance, and unidimensionality [10, 11]. At baseline, we also recorded basic demographic and clinical characteristics (as listed in Table 1). The 6-minute walk test (6MWT) was performed at baseline and follow-up in a 25-m long corridor in Newcastle and 20-m long corridor in London. Per currently agreed procedures [4], patients received feedback every minute of the current test time (i.e., the time left of the total 6-m test time).

**Table 1:** Baseline demographic and clinical characteristics of the patient sample

	Total sample (*n* = 150)	Lower total score (i.e., worsened ability) at follow-up (*n* = 65)	Same total score at follow-up (*n* = 49)	Higher total score (i.e., improved ability) at follow-up (*n* = 36)	*p* value†
Age, mean (SD) years	45 (14)	50 (12)	39 (16)	46 (11)	0.001
Sex, female	78 (52%)	38 (58%)	24 (49%)	16 (44%)	0.352
Age at first symptoms, mean (SD) years^a^	26 (17)	28 (18)	23 (17)	27 (16)	0.343
Disease duration, mean (SD) year^b^	20 (12)	22 (13)	16 (12)	20 (11)	0.090
Type of DM1^c^					
Congenital	8 (5%)	2 (3%)	5 (11%)	1 (3%)	0.141
Classical	101 (70%)	45 (70%)	29 (64%)	27 (75%)	0.583
Late onset	36 (25%)	17 (27%)	11 (24%)	8 (22%)	0.888
Part-time wheelchair dependency	22 (15%)	10 (15%)	4 (8%)	8 (22%)	0.190
6-minute walk test					
Baseline result, mean (SD) meters^d^	422 (152)	407 (129)	489 (155)	375 (137)	0.002
Follow-up result, mean (SD) meters^e^	404 (157)	356 (149)	491 (151)	368 (132)	< 0.001
Δ (follow-up—baseline result)^e^	− 30 (71)	− 52 (82)	− 14 (57)	− 14 (56)	0.015
Muscular impairment rating scale (MIRS) score					
I	19 (13%)	7 (11%)	9 (18%)	3 (8%)	0.323
II	40 (27%)	15 (23%)	20 (41%)	5 (14%)	0.015
III	30 (20%)	15 (23%)	8 (16%)	7 (19%)	0.669
IV	47 (31%)	22 (34%)	9 (18%)	16 (44%)	0.032
V	14 (9%)	6 (9%)	3 (6%)	5 (14%)	0.477
Education, mean (SD) years completed^d^	15 (3)	15 (3)	16 (3)	15 (3)	0.076
Current occupation					
Employed	61 (41%)	22 (34%)	26 (53%)	13 (36%)	0.096
Retired	22 (15%)	10 (15%)	7 (14%)	5 (14%)	0.975
Long-term sick leave	35 (23%)	17 (26%)	5 (10%)	13 (36%)	0.016
Unemployed/other	32 (21%)	16 (25%)	11 (22%)	5 (14%)	0.440
Follow-up, mean (SD) days	383 (47)	382 (40)	393 (50)	373 (54)	0.202

Data presented as *n* (%), if not specified otherwise. Total sample size: ^a^*n* = 144; ^b^*n* = 143; ^c^*n* = 145; ^d^*n* = 140; and ^e^*n* = 136. Because of rounding, percentages might not add up to 100% exactly

*DM1* myotonic dystrophy type 1

†Comparing patients with a higher DM1-Activ^C^ total score, same score, and lower score at follow-up

### Genetic analysis

Recent research [12] has shown that the length of the repeat expansion at birth as expressed by the progenitor allele is the most relevant predictor of disease onset and severity later in life, while disease progression is closely related to the rate of somatic expansion over time within different tissues (approximated as the difference between modal length at the time of DNA sampling and the progenitor allele, where the modal allele length is the most common repeat length in that tissue at time of sampling). For our analysis, to allow comparison of data, we included both CTG repeat counts from blood DNA (i.e., the estimated progenitor and modal allele length). CTG repeat length was estimated from blood DNA by the small-pool PCR assay as described by Gomes-Pereira et al. [13] using the CTG repeat-flanking primers DM-C and DM-DR [12, 14]. Replicate reactions were separated by gel electrophoresis, Southern blotted and hybridised using a ^32^P-labelled 56 × CTG repeat probe. Bands were detected by autoradiography and sized by comparison against the DNA molecular weight marker, using CLIQS software (TotalLab UK Ltd.). The bottom edge of the expanded allele bands was used to determine the estimated progenitor allele length [12]. The densest part of the expanded allele bands was used to estimate the modal allele length at the time of DNA sampling (i.e., CTG modal alleles). The CTG repeat length analysis was only available for patients recruited via Newcastle University, Newcastle upon Tyne.

### Statistical analysis

We calculated the distribution of replies across all items and levels within the DM1-Activ^C^ and the corresponding mean item scores, ranging from 0 (“Not possible to perform”) to 2 (“Possible, without any difficulty”), as well as the mean transformed total instrument score (ranging between 0 and 100, where a higher/lower score indicates a higher/lower ability to perform activities of daily living) at baseline and the follow-up visit. We related the total score to two previously derived [7] threshold values (amended for the transformed scale): ≤ 30 (indicating severe limitations in activities of daily living), and > 70 (indicating relatively few limitations). We calculated the unadjusted mean change in DM1-Activ^C^ total scores from baseline to follow-up for the total sample, as well as for patients with a higher and lower score at the follow-up visit, respectively. To account for differences in follow-up length, we also derived unadjusted mean annual changes in DM1-Activ^C^ total scores (assuming a linear change over time in DM1-Activ^C^ total scores at the patient level). We compared changes in DM1-Activ^C^ total scores by sex and type of DM1 defined based on age at onset of disease [congenital: ≤ 11 months; classical: 12 months–40 years; and late adult: > 40 years, with a Muscular Impairment Rating Scale (MIRS) score < III, and < 150 CTG repeats] using Welch’s *t* test. We also compared demographic and clinical characteristics of patients with a lower DM1-Activ^C^ total score at the follow-up (indicating a decreased ability to perform activities of daily living), a higher score (indicating an increased ability), and the same score using Welch’s analysis of variance (ANOVA) models and Pearson’s Chi-square test. We estimated Pearson’s correlation coefficients to investigate the crude relationship between disease duration (measured from onset), CTG repeat length, MIRS score, and 6MWT result, respectively, and annual changes in DM1-Activ^C^ total scores. In addition, to further investigate the association between the baseline DM1-Activ^C^ total score (which may serve as a meaningful and easily measurable criterion for future clinical trials) and annual changes in total scores, we fitted an ordinary least squares linear regression model to our study data, with annual change in the DM1-Activ^C^ total score as the dependent variable and the baseline score as the primary explanatory variable. To adjust for confounding effects, the model was specified to also include sex, age, and estimated progenitor CTG repeat length, as well as an interaction variable between estimated progenitor CTG repeat length (normalised by log transformation) and age, per previous research [12]. Robust standard errors were derived using the Huber/White/sandwich estimator. All analyses were conducted in Stata 14 (StataCorp, College Station, TX, USA).

## Results

A total of *n* = 213 patients with DM1 met all study inclusion criteria and were enrolled into the PhenoDM1 study. Of these, *n* = 150 completed the DM1-Activ^C^ in accordance with instructions at both the baseline and follow-up visit. Demographic and clinical characteristics of the sample are presented in Table 1. At baseline, less than half (41%, 61 of 150) of all patients reported being employed (part-time or full-time) and one-third (33%, 43 of 132) of those of working age (i.e., < 65 years) were retired or on long-term sick leave. Mean estimated progenitor and modal allele length, based on data for 56% (84 of 150) and 55% (82 of 150) of patients, respectively, were 234 CTG repeats (SD: 171, range: 55–916) and 465 CTG repeats (344, 62–1441). In the subset of patients from the PhenoDM1 study analyzed as part of this work, three had variant repeat interruptions.

At baseline, the mean DM1-Activ^C^ total score in our sample was estimated at 71.24 [SD: 21.53, range: 28–100, 95% confidence interval (CI) 67.77–74.71]. Approximately 1% (2 of 150) scored ≤ 30 (indicating severe limitations in activities of daily living), and 48% (72 of 150) scored > 70 (indicating relatively few limitations). At follow-up, after a mean duration of 383 days, the mean DM1-Activ^C^ total score in our sample was estimated at 69.04 (SD: 21.67, range: 25–100, 95% CI 65.54–72.54), with the same proportions scoring ≤ 30 and > 70.

The unadjusted mean change in the DM1-Activ^C^ total score during follow-up was estimated at − 2.20 (SD: 9.50, range: − 47 to 28, 95% CI − 3.73 to − 0.67). Approximately 43% (65 of 150) of patients had a lower score at the follow-up visit (indicating a decreased ability to perform activities of daily living), 24% (36 of 150) a higher score (indicating an increased ability), and 33% (49 of 150) the same score at baseline and follow-up. The mean unadjusted change in patients with a lower total score at follow-up was − 9.68 (SD: 7.74, range: − 47 to − 1, 95% CI − 11.60 to − 7.76), and a higher total score 8.31 (SD: 7.13, range: 1–28, 95% CI 5.89–10.72). Demographic and clinical characteristics of the three patient groups are presented in Table 1.

**Fig. 1:**
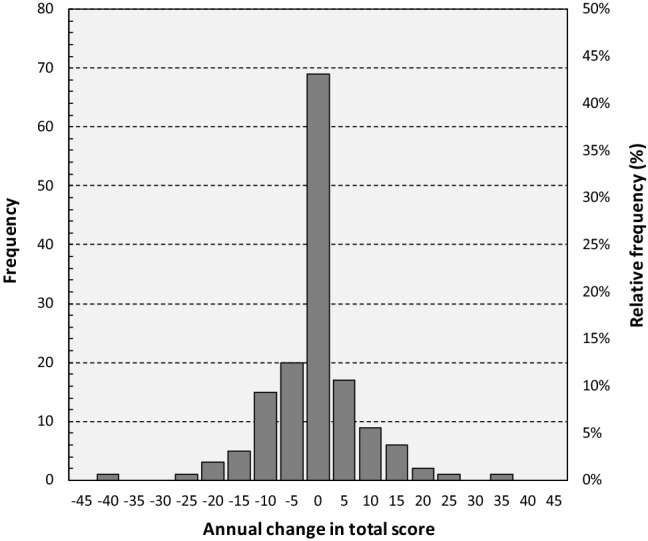
Histogram of annual changes in DM1-Activ^C^ total scores. A higher/lower score indicates a higher/lower ability to perform activities of daily living

Accounting for the patient-specific length of follow-up, and assuming a linear change (at the patient level) in DM1-Activ^C^ total scores, the unadjusted mean annual change in the DM1-Activ^C^ total score in the total sample population was − 2.06 (SD: 9.14, range: − 43.57 to 34.55, 95% CI − 3.54 to − 0.59) (Fig. 1). The corresponding estimate for patients with a lower total score at follow-up was − 9.25 (SD: 7.26, range: − 43.57 to − 0.98, 95% CI − 11.05 to − 7.45), and a higher score 8.10 (SD: 7.14, range: 0.90–34.55, 95% CI 5.68–10.51). We found no significant differences between women and men in the unadjusted mean annual change in the DM1-Activ^C^ total score across follow-up (− 2.24 vs. − 1.87, *p* = 0.808). The unadjusted mean annual change in the DM1-Activ^C^ total score was − 1.96 (SD: 9.25, range: − 43.57 to 24.64, 95% CI − 3.79 to − 0.13) for patients with classical DM1, − 1.48 (SD: 7.32, range: − 17.18 to 9.13, 95% CI − 7.61 to 4.64) for congenital DM1, and − 2.69 (SD: 9.95, range: − 20.93–34.55, 95% CI − 6.06 to 0.68) for late onset DM1 (classical vs. congenital: *p* = 0.887; classical vs. late onset: *p* = 0.690; and congenital vs. late onset: *p* = 0.748). In patients with variant repeat interruptions (*n* = 3), the mean annual change in the DM1-Activ^C^ total score was − 2.63 (SD: 7.54, range: − 5.09–9.99, 95% CI − 16.12–21.37). Changes in scores were not significantly different across categories of current occupation (all *p* > 0.479).

Table 2 summarizes changes during follow-up to the individual items within the DM1-Activ^C^ for the total sample, as well as patients with a higher total score (i.e., an improved ability to perform activities of daily living) and lower total score (i.e., a worsened ability) at follow-up, respectively. For example, 86% (31 of 36) of patients who had a higher total score at the end of the follow-up provided the same answer at the baseline and follow-up visit to the question pertaining to their ability to eat soup. As shown in the table, replies to most items did not change from the baseline to the follow-up visit. For the subgroup with a higher total score at follow-up, the item with the largest positive change (i.e., an increased score from 0 to 1, or 1 to 2) was “Carry and put down heavy object (10 kg)”, where 44% (16 of 36) of patients indicated improvement in their ability, followed by “Serve coffee/tea on a tray” (42%, 15 of 36), and “Stand on one leg” (36%, 13 of 36). Worsened ability (i.e., a decreased item score from 2 to 1, or 1 to 0) for this subgroup of patients was first and foremost noted for “Stand up from squatting position” (17%, 6 of 36), and “Dress your lower body” and “Wash your upper body” (14%, 5 of 36, respectively). For patients with a lower total score at follow-up, the largest improvement was noted for “Stand up from squatting position”, where 11% (7 of 65) had a higher item score, followed by “Walk three flights of stairs” and “Carry and put down heavy object (10 kg)” (8%, 5 of 65, respectively). The largest loss in ability for this subgroup concerned “Stand up from squatting position”, where 38% (25 of 65) of patients had a lower item score at follow-up, followed by “Dance” (34%, 22 of 65). In the total sample, the item exhibiting the largest positive change was “Carry and put down heavy object (10 kg)”, where 19% (28 of 150) had a higher item score at follow-up, followed by “Stand up from squatting position” (14%, 21 of 150), and “Serve coffee/tea on a tray” and “Stand on one leg” (12%, 18 of 150, respectively). The item with the largest negative change was “Stand up from squatting position”, where 23% (34 of 150) had a lower item score at follow-up, followed by “Stand on one leg” (20%, 30 of 150), and “Tie the laces of your shoes” and “Walk uphill” (18%, 27 of 150, respectively).

**Table 2:** Changes from baseline to follow-up to individual items within the DM1-Activ^C^

	Total sample (*n* = 150)			Lower total score (i.e., worsened ability) at follow-up (*n* = 65)			Higher total score (i.e., improved ability) at follow-up (*n* = 36)		
	No item change	Higher item score at follow-up	Lower item score at follow-up	No item change	Higher item score at follow-up	Lower item score at follow-up	No item change	Higher item score at follow-up	Lower item score at follow-up
Eat soup	137 (91%)	7 (5%)	6 (4%)	58 (89%)	3 (5%)	4 (6%)	31 (86%)	3 (8%)	2 (6%)
Visit family or friends	129 (86%)	10 (7%)	11 (7%)	56 (86%)	2 (3%)	7 (11%)	26 (72%)	8 (22%)	2 (6%)
Care for your hair and body	129 (86%)	8 (5%)	13 (9%)	53 (82%)	2 (3%)	10 (15%)	29 (81%)	4 (11%)	3 (8%)
Dress your lower body	125 (83%)	7 (5%)	18 (12%)	52 (80%)	1 (2%)	12 (18%)	26 (72%)	5 (14%)	5 (14%)
Wash your upper body	128 (85%)	10 (7%)	12 (8%)	55 (85%)	3 (5%)	7 (11%)	27 (75%)	4 (11%)	5 (14%)
Take a shower	124 (83%)	12 (8%)	14 (9%)	53 (82%)	2 (3%)	10 (15%)	27 (75%)	7 (19%)	2 (6%)
Wash your lower body	127 (85%)	9 (6%)	14 (9%)	53 (82%)	1 (2%)	11 (17%)	27 (75%)	6 (17%)	3 (8%)
Get out of bed	120 (80%)	11 (7%)	19 (13%)	51 (78%)	0 (0%)	14 (22%)	29 (81%)	6 (17%)	1 (3%)
Move a chair	116 (77%)	14 (9%)	20 (13%)	52 (80%)	1 (2%)	12 (18%)	24 (67%)	9 (25%)	3 (8%)
Do the dusting/cleaning	126 (84%)	9 (6%)	15 (10%)	52 (80%)	2 (3%)	11 (17%)	30 (83%)	6 (17%)	0 (0%)
Do the shopping	126 (84%)	10 (7%)	14 (9%)	55 (85%)	0 (0%)	10 (15%)	27 (75%)	7 (19%)	2 (6%)
Tie the laces of your shoes	112 (75%)	11 (7%)	27 (18%)	42 (65%)	2 (3%)	21 (32%)	27 (75%)	7 (19%)	2 (6%)
Catch an object (e.g., a ball)	118 (79%)	16 (11%)	16 (11%)	48 (74%)	4 (6%)	13 (20%)	28 (78%)	6 (17%)	2 (6%)
Use dustpan and brush	111 (74%)	13 (9%)	26 (17%)	45 (69%)	2 (3%)	18 (28%)	22 (61%)	10 (28%)	4 (11%)
Empty dustbin	117 (78%)	12 (8%)	21 (14%)	46 (71%)	1 (2%)	18 (28%)	24 (67%)	10 (28%)	2 (6%)
Make up your bed	111 (74%)	14 (9%)	25 (17%)	47 (72%)	3 (5%)	15 (23%)	24 (67%)	9 (25%)	3 (8%)
Vacuum clean	119 (79%)	15 (10%)	16 (11%)	50 (77%)	1 (2%)	14 (22%)	27 (75%)	9 (25%)	0 (0%)
Serve coffee/tea on a tray	111 (74%)	18 (12%)	21 (14%)	51 (78%)	0 (0%)	14 (22%)	20 (56%)	15 (42%)	1 (3%)
Dance	107 (71%)	17 (11%)	26 (17%)	40 (62%)	3 (5%)	22 (34%)	26 (72%)	8 (22%)	2 (6%)
Stand up from squatting position	95 (63%)	21 (14%)	34 (23%)	33 (51%)	7 (11%)	25 (38%)	21 (58%)	9 (25%)	6 (17%)
Stand on one leg	102 (68%)	18 (12%)	30 (20%)	44 (68%)	1 (2%)	20 (31%)	20 (56%)	13 (36%)	3 (8%)
Walk uphill	115 (77%)	8 (5%)	27 (18%)	44 (68%)	1 (2%)	20 (31%)	29 (81%)	6 (17%)	1 (3%)
Walk three flights of stairs	115 (77%)	15 (10%)	20 (13%)	46 (71%)	5 (8%)	14 (22%)	26 (72%)	8 (22%)	2 (6%)
Carry and put down heavy object (10 kg)	96 (64%)	28 (19%)	26 (17%)	39 (60%)	5 (8%)	21 (32%)	17 (47%)	16 (44%)	3 (8%)
Run	116 (77%)	16 (11%)	18 (12%)	46 (71%)	4 (6%)	15 (23%)	24 (67%)	11 (31%)	1 (3%)

Data reported as *n* (%). Because of rounding, percentages might not add up to 100% exactly. In total, *n* = 49 patients had the same DM1-Activ^C^ total score at baseline and the follow-up visit. A higher/lower score indicates a higher/lower ability to perform activities of daily living

We found the annual change in the DM1-Activ^C^ total score to be related to the total score at baseline (i.e., patients’ baseline ability to perform activities of daily living) (*p* = − 0.20, *p* = 0.015), but not estimated progenitor or modal allele CTG repeat length (*p* = 0.937 and *p* = 0.897, respectively), disease duration (*p* = 0.712), or baseline MIRS or 6MWT result (*p* = 0.901 and *p* = 0.567, respectively). Results from our regression analysis revealed that each ten-point change in the DM1-Activ^C^ total score at baseline was associated with a mean annual total score change of − 1.37 (SE: 0.66, 95% CI − 2.68 to − 0.06, *p* = 0.041) when adjusting for age, sex, and estimated progenitor CTG repeat length (full model results available as supplemental material online).

## Discussion

The objective of this study was to investigate change over time in ability to perform activities of daily living as measured using the DM1-Activ^C^ in patients with DM1. Taken together, our results reveal substantial heterogeneity in changes across follow-up in DM1-Activ^C^ total scores at the population level. Indeed, almost half of our sample (43%) had a lower score at the follow-up visit (indicating a decreased ability to perform activities of daily living), 24% a higher score (indicating an increased ability), and 33% the same score at baseline and follow-up. Results from the 6MWT were distributed in line with these findings, showing a markedly greater decline for those with a lower DM1-Activ^C^ total score at the follow-up. In addition, we found baseline ability to perform activities of daily living to be significantly associated with annual change in total scores, but not sex, disease duration, timed test results, or CTG repeat length. These data should be helpful to inform the design of future studies based on the DM1-Activ^C^.

Comparing our results with previous research, changes in DM1-Activ^C^ total scores have been estimated and reported as part of the OPTIMISTIC trial in patients with DM1 with severe fatigue [8]. In that study, the unadjusted mean change in the total score across follow-up (comprising 10 months) for patients receiving cognitive behavioral therapy with optional graded exercise therapy (*n* = 128; mean age: 45 years; 45% female) was estimated at 2.70. For patients receiving standard of care (*n* = 127; mean age: 46 years; 47% female), the mean change was − 2.21. Adjusting our estimates to reflect the same follow-up duration, the unadjusted mean change in the DM1-Activ^C^ total score in our sample was − 1.72 (95% CI − 2.95 to − 0.49). However, it should be emphasized that these studies are not directly comparable due to non-trivial differences concerning demographic and clinical characteristics of the sample populations.

We found that replies to only a limited set of items changed from baseline to end of follow-up (Table 2). Specifically, in the total sample, replies to only three individual items changed during follow-up for > 30% of patients, and 15 items for > 20%. Moreover, changes to replies to all but two items, namely “Eat soup” and “Carry and put down heavy object (10 kg)”, were indicative of lost ability (i.e., a net negative change in item scores). Notable examples of items for which a larger proportion of patients with any change had a lower as opposed to higher score at follow-up include “Walk uphill” (18% vs. 5%), “Tie the laces of your shoes” (18% vs. 7%) and “Stand up from squatting position” (23% vs. 14%). Looking at the subset of patients with a higher total score at follow-up, items with the largest positive change were “Carry and put down heavy object (10 kg)”, “Serve coffee/tea on a tray”, and “Stand on one leg”. To our surprise, 44% of these patients indicated an improvement in their ability to “Carry and put down heavy object (10 kg)” across follow-up. Furthermore, for this subgroup, the only items where > 10% of patients had a lower score at follow-up were “Stand up from squatting position”, “Use dustpan and brush”, “Dress your lower body”, and “Wash your upper body”. The items with the largest negative change in the group of patients with a lower total score at follow-up were “Stand up from squatting position” and “Dance”. Indeed, for these two items, more than one-third of patients had a lower item score at the end of follow-up. Only one item (“Stand up from squatting position”) had a positive change in > 10% of patients in this subgroup. Taken together, these data show that change over time in ability to perform activities of daily living as recorded using the DM1-Activ^C^ is highly variable in DM1, and that changes (negative, as well as positive) seem to involve gross and fine motor skills of both the upper and lower extremities. In terms of measuring annual change, our analysis also reveals that some items appear to be lacking relevance at the population level with respect to the underlying, highly variable natural evolution of the disease. More research is needed to fully understand these properties of the DM1-Activ^C^.

The minimal clinically important difference in DM1-Activ^C^ total scores is yet to be estimated. For this reason, it is not straightforward to interpret the magnitude of the annual changes reported as part of this work. Still, considering that a one-point change at the item level means that a specific task no longer can be performed without difficulty, or at all, and given the fact that items within the DM1-Activ^C^ captures common activities of daily living involving significant muscle function, it could be argued that even a one-point change would be meaningful from the perspective of the individual patient.

As mentioned in brief above, results from our adjusted regression analysis showed that the baseline DM1-Activ^C^ total score was significantly associated with the annual change in total score. Specifically, we found that patients with a higher score at baseline had a larger negative change across follow-up when controlling for age, sex, and estimated progenitor CTG repeat length. Although a diminishing marginal loss in ability in relation to the initial level of ability may appear intuitive (i.e., the more ability you possess, the more you can theoretically lose over time), this result would be expected to be of importance when designing trials involving the DM1-Activ^C^. In particular, our data suggests that it may be challenging to conduct meaningful measurement of changes in ability to perform activities of daily living over time using the DM1-Activ^C^ in patient populations characterized by a low starting level of ability. That being said, it is important to keep in mind that additional research is needed to confirm our findings, preferably in a larger sample with an even greater diversity of ability at baseline.

The main limitation of our study concerns the precision and external validity of our results due to the relatively small number of patients in comparison with research of more common illnesses. Yet, it should be noted that our sample of 150 patients is large in the context of studies in DM1 [15]. In addition, the DM1-Activ^C^ data may be subject to bias due to, for example, incorrect reporting. This issue would be expect to be of particular relevance in populations characterized by some degree of cognitive impairment, such as DM1. However, per the study procedures, patients with significant cognitive impairment were not eligible to participate, which should help minimize errors associated with the data collection. Another potential source of variability across follow-up includes learning effects, where patients’ experience of the DM1-Activ^C^ at baseline may influence their assessment at follow-up [4, 16]. We were also unable to unable to infer causality behind identified associations due to the observational nature of our data. Finally, when interpreting our regression analysis results, it is important to keep in mind that the estimated coefficients may be subject to unmeasured confounding.

In conclusion, we show that change over time in ability to perform activities of daily living as recorded through the DM1-Activ^C^ varies substantially between patients with DM1. Our data contribute to the understanding of the natural evolution of the disease, and should be helpful to inform the design of future trials based on the DM1-Activ^C^.

## Electronic supplementary material

Below is the link to the electronic supplementary material.

Supplementary file1 (DOCX 23 kb)

## Data Availability

The data that support the findings of this study are not publicly available due to privacy or ethical restrictions.
