# Supplementary material for: Change over time in ability to perform activities of daily living in myotonic dystrophy type 1
Source: J Neurol. 2020 Jun 15;267(11):3235–42. doi: 10.1007/s00415-020-09970-6 (PMC7578145; doi:10.1007/s00415-020-09970-6)
Supplement: Supplementary file 1 — Supplementary file1 (DOCX 23 kb) [file 415_2020_9970_MOESM1_ESM.docx]

**Supplemental Material**

**Table e1: Regression analysis of annual change in the DM1-Activ^C^ total score**

| **Independent variable** | **Coefficient** | **SE** | **t** | **p-value** | **95% CI** |
| --- | --- | --- | --- | --- | --- |
| DM1-Active^C^ total baseline score | -0.14 | 0.07 | -2.08 | 0.041 | -0.27 to -0.01 |
| Sex, female | -0.23 | 2.04 | -0.11 | 0.909 | -4.30 to 3.83 |
| Age, in years | 0.36 | 0.35 | 1.03 | 0.305 | -0.34 to 1.07 |
| Estimated progenitor CTG repeat length | 0.86 | 3.54 | 0.24 | 0.809 | -6.19 to 7.90 |
| Age*Estimated progenitor CTG repeat length | -0.12 | 0.07 | -1.82 | 0.072 | -0.26 to 0.01 |
| Constant | 14.95 | 22.81 | 0.66 | 0.514 | -30.46 to 60.37 |

*Note: Dependent variable: annual change in the DM1-Activ^C^ total score. Sample size: n = 84. Prob. > F = 0.026. R^2^ =0.160. Estimated progenitor CTG repeat length was normalised by log transformation. Standard error (SE). Confidence interval (CI).*
